# Targeting p66Shc to restore K_ATP_ channel and renal microvascular responses in a preclinical model of diabetic nephropathy

**DOI:** 10.3389/fphys.2025.1620591

**Published:** 2025-08-05

**Authors:** Bradley Miller, John Imig, Samaneh Goorani, Perrin Schupbach, Franklin Hays, Doris M. Benbrook, Andrey Sorokin

**Affiliations:** ^1^Department of Medicine, Medical College of Wisconsin, Milwaukee, WI, United States; ^2^Drug Discovery Center, Department of Pharmacology and Toxicology, Medical College of Wisconsin, Milwaukee, WI, United States; ^3^Department of Pharmaceutical Sciences, University of Arkansas for Medical Sciences, Little Rock, AR, United States; ^4^Harold Hamm Diabetes Center, Stephenson Cancer Center, Department of Nutritional Sciences, College of Allied Health, University of Oklahoma Health Sciences, Oklahoma, OK, United States; ^5^Harold Hamm Diabetes Center, Stephenson Cancer Center, Department of Obstetrics and Gynecology, University of Oklahoma Health Sciences Center, Oklahoma, OK, United States

**Keywords:** adaptor protein, diabetic nephropathy, rat model, vasculature, autoregulation

## Abstract

Renal microvascular injury occurs in most patients with diabetes, representing one of the main causes underlying chronic kidney disease development. We have previously published that overexpression of adaptor protein p66Shc is implicated in the loss of renal microvascular reactivity in rats with diabetic nephropathy (DN) induced by injection of streptozotocin (STZ). Since sulfur heteroarotinoid A2 (SHetA2) is known to interfere with p66Shc signaling, we tested whether SHetA2 would restore renal microvascular reactivity and mitigate kidney injury in our rat model of DN. Dahl salt sensitive wild-type (SS) and p66Shc knockout rats were used in a well-established rat model of DN, characterized by progressive proteinuria, hyperfiltration, and display of renal histological lesions. SHetA2 was either added acutely to isolated rat afferent arterioles or chronically administrated to rats during DN development. The ability of SHetA2 treatment to restore afferent arteriolar contraction in response to increased perfusion pressure or ATP was evaluated using the perfused juxtamedullary nephron preparation. The progression of renal damage was evaluated by measuring urinary protein excretion. Comparison of renal microvascular responses to perfusion pressure in p66Shc knockout rats and parental SS rats, in the presence and absence of acute preincubation with SHetA2, revealed ability of SHetA2 to restore renal microvascular reactivity in SS rats with little effect upon p66Shc knockouts. Moreover, chronic treatment with SHetA2 prevented loss of renal microvascular responses. Even though targeting p66Shc with SHetA2 restored renal afferent arteriolar reactivity caused by DN, it had limited effect upon a biomarker of renal injury. Thus, additional studies are necessary to develop SHetA2 for prevention and treatment of DN-induced kidney damage.

## 1 Introduction

The global diabetes prevalence in 2021 in adults (20–79 years) was estimated to be 10.5% (536.6 million people), rising to 12.2% (783.2 million) in 2045 (Sun et al., 2022). DN is one of the most severe complications of diabetes and is the leading cause of end-stage renal disease in the world (Samsu, 2021). Renal vascular dysfunction associated with DN results in diabetic hyperfiltration which causes irreversible nephron damage (Tonneijck et al., 2017). It is generally accepted that diabetic hyperfiltration is promoted by undesirable changes in afferent arteriolar vascular tone, causing vasodilation (Carmines, 2010). Increased expression of adaptor protein p66Shc, a signaling molecule from Shc family of adaptor proteins, has been associated with progression of DN (Mousavi et al., 2022). We have previously reported that inactivation of the adaptor protein p66Shc decreases afferent arteriolar K_ATP_ channel activity and prevents renal and microvascular damage in diabetic salt-sensitive (SS) rats (Miller et al., 2018). Our preliminary data provide rationale to focus on protein p66Shc, as a promoter of hyperfiltration and potential target for novel therapeutic interventions in DN (Miller et al., 2018; Wright et al., 2018). The uniqueness of the diabetic Dahl SS rat compared to other models of STZ-induced diabetes, is the development of comparable functional and morphological renal abnormalities observed in patients with DN (Pourghasem et al., 2015; Brosius et al., 2009; Slaughter et al., 2013). It has been established that afferent arterioles of hypertensive SS rats exhibit impaired myogenic response to increased perfusion pressure. Since sulfur heteroarotinoid A2 (SHetA2) modulates p66Shc signaling and restores renal microvascular responses in hypertensive SS rats (Miller et al., 2025), we tested whether SHetA2 would restore renal microvascular reactivity and mitigate kidney injury in our rat model of DN. Unique SS rat mutants, developed in our laboratory, with p66Shc deletion allowed us to verify whether SHetA2 effect is mediated through the modulation of p66Shc signaling.

## 2 Results

### 2.1 SHetA2 restores microvascular reactivity in renal afferent arterioles isolated from rats with induced DN

**FIGURE 1:**
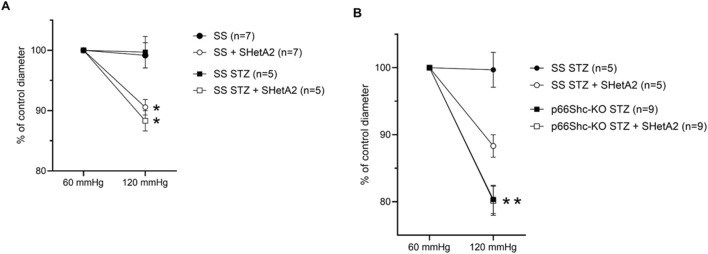
SHetA2 restored renal microvascular responses in diabetic rats measured in perfused juxtamedullary nephron preparation. Microvascular responses of renal afferent preglomerular arterioles to perfused pressure were compared in STZ-treated (squares) and control (circles) wild type male SS rats **(A)** in the presence (blank circles, n = 7 vessels from 3 rats; and squares, n = 5 vessels from 3 rats) and absence (black circles and black squares) of SHetA2. Vascular responses of afferent arterioles of STZ-treated p66Shc knockout rats **(B)** in the presence (blank square, n = 9 vessels from 4 rats) and absence of SHetA2 (black square). SHetA2 was added directly to isolated renal vessels. Values are expressed, relative to control diameters at 60 mmHg, as means ± SE. *P <0 .0001 SHetA2 treated vs control, **P = 0.001 p66Shc-KO STZ vs. SS STZ.

SS rats were rendered diabetic with streptozotocin as previously reported (Miller et al., 2018). These rats, when maintained on low salt diet, do not develop hypertension and do not have metabolic syndrome making them suitable to study type 1 diabetes after streptozotocin injection. However, Dahl SS rats have some vascular dysfunction at baseline (Ren et al., 2014) and have an impaired response to increased perfused pressure even on low salt diet (Takenaka et al., 1992). As we reported previously, microvascular renal responses in SS rats with induced DN are lost due to p66Shc-mediated signaling (Miller et al., 2018). Afferent arterioles of hypertensive SS rats exhibit impaired myogenic response to increased perfusion pressure, and this impairment has been shown in part to be the result of increased p66Shc expression in renal microvessels as p66Shc-KO rats have restored contractility and are protected from progressive proteinuria and renal injury (Miller et al., 2016). Furthermore, recent evidence suggests acute and chronic treatment with SHetA2 restores contractility in renal microvessels of hypertensive SS rats expressing p66Shc (Miller et al., 2025). By 6 weeks of STZ-induced hyperglycemia, afferent arterioles of SS rats lose myogenic tone, i.e., contraction, in response to higher intraluminal pressure. Interestingly, acute treatment of afferent arterioles with 2.5 µM SHetA2 restored contractility in response to increased pressure (Figure 1A), an effect also seen in SS rats with hypertensive nephropathy. Although knockout of p66Shc also restored contraction of vessels in rats with both HN and DN, acute treatment with SHetA2 did not provide an additive improvement of tone, suggesting that the action of SHetA2 may involve a p66Shc-dependent mechanism (Figure 1B).

### 2.2 Chronic administration of SHetA2 prevents loss of microvascular reactivity in rat model of diabetic nephropathy

**FIGURE 2:**
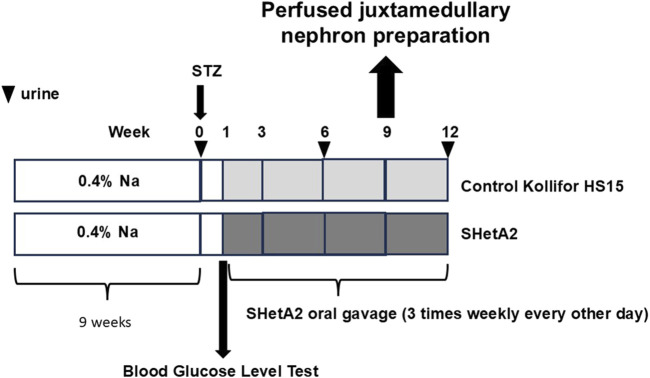
Schematic representation of experiments to establish ability of chronic administration of SHetA2 to restore renal microvascular responses in diabetic rats and prevent the progression of renal injury associated with type 1 diabetes. SHetA2 is administrated by oral gavage (3 times weekly every other day). Vehicle for SHetA2 is Kolliphor^®^ HS 15 (a potent non-ionic solubilizer and emulsifying agent, with low toxicity, which is ideal for solubilizing low-solubility actives in microemulsions). Urinary albumin excretion is measurement at indicated times (marked by triangles).

**FIGURE 3:**
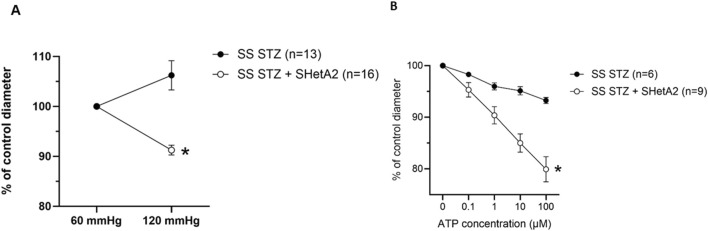
Chronic administration of SHetA2 restored renal microvascular responses in diabetic rats measured in perfused juxtamedullary nephron preparation. **(A)** Diameters of afferent arterioles were measured in response to increase in perfused pressure for SS STZ (black circles, n = 13 vessels from 7 rats) and SS STZ chronically treated with SHetA2 (white circles, n = 16 vessels from 6 rats). **(B)** Diameters of afferent arterioles were measured in response to ATP for SS STZ (black circles, n = 6 vessels studied from 3 rats) and SS STZ rats chronically treated with SHetA2 (white circles, n = 9 vessels studied from 4 rats). Values are expressed, relative to control diameters at 60 mmHg, as means ± SE. *P = 0.002 SHetA2 vs. control.

As shown in Figure 1, addition of SHetA2 to isolated renal afferent arterioles was sufficient to partially restore myogenic tone and reduce vessel dilation. Accordingly, we tested whether chronic SHetA2 administration would prevent p66Shc-induced vascular dysfunction in our rat model of Type 1 diabetes. The oral formulation and weekly dosing of SHetA2 required for maintaining the predicted effective drug dose were determined by us previously (Miller et al., 2025). Rats were maintained on 0.4% salt diet for 9 weeks prior to single STZ injection. To determine if SHetA2 was able to improve myogenic tone *in vivo*, SHetA2 was given to diabetic SS rats by oral gavage over 11 weeks (Figure 2). Blood glucose levels were checked to confirm the development hyperglycemia and following 9 weeks of SHetA2 treatment the perfused juxtamedullary nephron preparations were used to evaluate microvascular reactivity. Myogenic tone was improved upon treatment, as constriction of afferent arterioles in response to increased pressure was restored by SHetA2 treatment [Figure 3A, *P <0 .0001]. Purinergic signaling via P2X receptors is thought to be a mediator of renal resistance vessel constriction in response to increased intraluminal pressures to maintain proper blood flow to glomerular capillaries. In agreement, vessel contraction to acute ATP treatment was restored in diabetic SS rats treated chronically with SHetA2 (Figure 3B, F1, 13) = [14.10], *P = 0.002).

### 2.3 SHetA2 effect upon microvascular reactivity is coupled with inhibition of K_ATP_ channels, not with regulation of calcium influx

**FIGURE 4:**
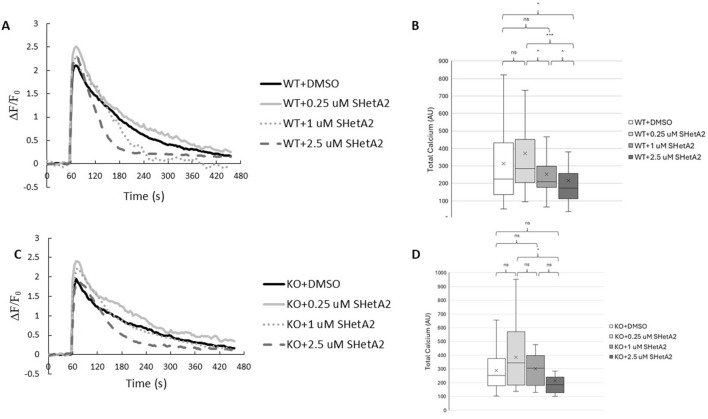
Effects of SHetA2 on Ca^+2^ mobilization in renal vascular SMCs. P66Shc-WT **(A)** and p66Shc-KO **(C)** SMCs were loaded with Fluo4-AM (2 µM) and incubated with specified concentrations of SHetA2 or 0.3% DMSO as a control. Baseline Fluo-4 fluorescence was measured for 1 min prior to stimulation with ET-1 (100 nM). Responses were normalized to basal fluorescence levels. Boxplots for corresponding total Ca^+2^ measurements, calculated as integrals of the fluorescent responses, are shown for WT **(B)** and p66Shc-KO **(D)** SMCs. P66Shc-WT: DMSO (n = 68), 0.25 µM SHetA2 (n = 35), 1 µM SHetA2 (n = 42), 2.5 µM SHetA2 (n = 51). P66Shc-KO: DMSO (n = 13), 0.25 µM SHetA2 (n = 16), 1 µM SHetA2 (n = 10), 2.5 µM SHetA2 (n = 11). ns = not statistically significant, ^ = p < 0.1, * = p < 0.05, *** = p < 0.001.

We have previously reported that p66Shc-mediated loss of vascular dysfunction in rats with hypertension-induced nephropathy is associated with inhibition of calcium influx in renal vascular smooth muscle cells (SMC) (Miller et al., 2016). To test whether SHetA2 effect on myogenic tone of rats with induced DN is caused by modulating calcium influx, we have determined the effects of SHetA2 on calcium mobilization in renal vascular SMCs. When SMCs were stimulated with vasoactive peptide endothelin-1 (ET-1), treatment of cells with 2.5 µM SHetA2 reduced calcium entry compared to the DMSO control (Figures 4A,B). In addition, the effect of SHetA2 on calcium was independent of p66Shc, as 2.5 µM SHetA2 had the same inhibitory effect on p66Shc-KO SMCs (Figures 4C,D). For both strains of SMC, 0.25 and 1 µM SHetA2 did not have a significant impact on calcium mobilization. Our data suggest that SHetA2-mediated restoration of renal vascular tone cannot be explained by increased calcium mobilization (Figure 4).

**FIGURE 5:**
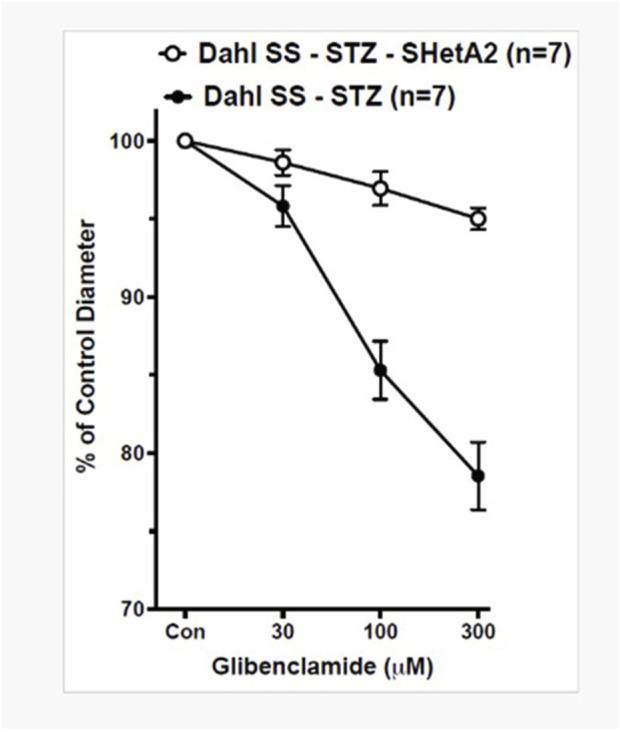
SHetA2 inhibits the activity of K_ATP_ channels in diabetic rats. The diameters of afferent arterioles were measured from STZ-treated SS rats chronically treated with (white circles, n = 7 vessels from 3 rats) or without (black circles, n = 7 vessels from 3 rats) SHetA2 in response to acute application of K_ATP_ channel inhibitor glibenclamide. The contraction of vessels indicates the presence of active KATP channels responsible for vasodilation causing hyperfiltration of the glomerulus. Values are expressed, relative to untreated diameters, as means ± SE. *P <0 .001 SHetA2 vs. control.

K_ATP_ channels are present in SMC, and by modulating vascular responses to diverse stimuli, they contribute to physiological regulation of vascular tone (Brayden, 2002). Afferent arteriolar dilation and glomerular hyperfiltration in diabetes are due to increased K_ATP_ channel availability and activity. We have previously reported that inactivation of p66Shc decreases afferent arteriolar K_ATP_ channel activity and decreases renal damage in diabetic SS rats (Miller et al., 2018). The antidiabetic drug glibenclamide binds and inhibits K_ATP_ inhibitory regulatory subunit resulting in cell membrane depolarization and an increase in intracellular calcium and vessel contraction. We show here that while the dose-dependent inhibition of K_ATP_ channels by glibenclamide results in constriction of afferent arterioles of SS STZ-treated rats, there is no effect upon SS STZ-treated rats that received SHetA2 every other day (Figure 5 F1 12) = [27.31], *P <0 .001). This result indicates that SHetA2 treatment reduces K_ATP_ channel activity implicated in hyperfiltration of the glomerulus during diabetes. This is in agreement with our previous finding in STZ-treated p66Shc-KO rats (Mousavi et al., 2022).

### 2.4 SHetA2 has limited effect upon DN-induced kidney damage

**FIGURE 6:**
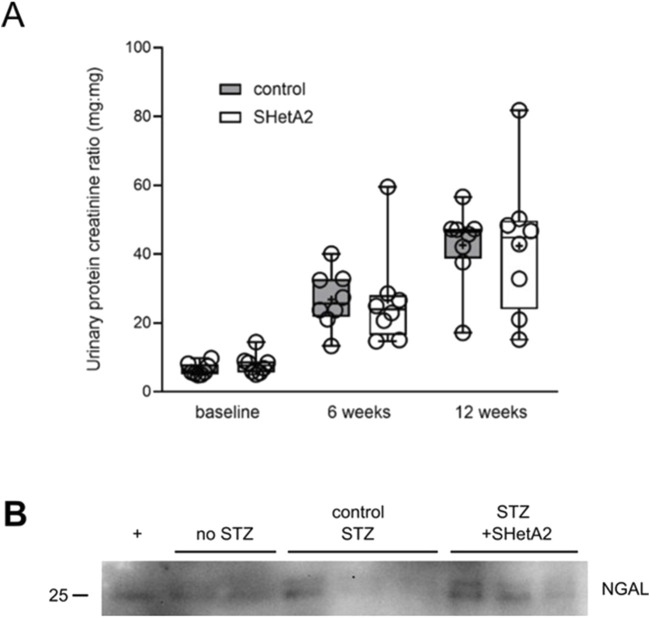
Proteinuria of STZ-induced, diabetic SS rats. **(A)** Urine was collected before (baseline), 6 weeks and 12 weeks after induction of hyperglycemia. Control SS (gray) and SS rats chronically treated with SHetA2 (white). Tukey’s box plot represents 25th quartile, median (horizontal line), and 75th quartile; whiskers denote max and min data and “+” denotes mean. **(B)** Tubular injury marker NGAL was assessed in urine of diabetic SS rats. + denotes positive control (Miller et al., 2025).

Progressive renal decline is the major feature of DN in type 1 diabetes (Krolewski et al., 2014). The most characteristic marker associated with DN progression is proteinuria (Lin et al., 2018). We evaluated the effect of chronic treatment with SHetA2 upon proteinuria in rats with induced DN. Despite the improvement of myogenic tone, we did not detect the inhibition of progressive proteinuria seen in DN with chronic treatment using SHetA2 up to 12 weeks (Figure 6A F2 28) = [0.03], P = 0.97). Urinary Neutrophil gelatinase-associated lipocalin (NGAL) has been associated with tubular injury in the progression of DN (Alter et al., 2012; Liu et al., 2015). In agreement with our proteinuria results, SHetA2 treatment of diabetic rats did not modify the levels of NGAL in the urine as assessed by Western blot analysis (Figure 6B).

### 2.5 SHetA2 effect on p66Shc-mediated ROS activity

**FIGURE 7:**
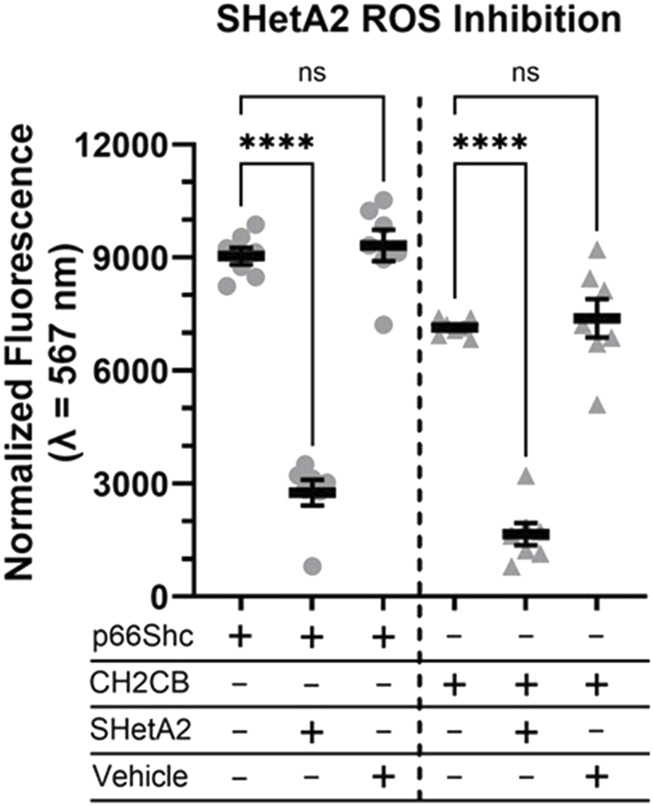
ROS producing activity of purified human p66Shc is attenuated in the presence of SHetA2. Recombinant human full-length p66Shc (grey circles), or the N-terminal ROS producing CH2CB domain (grey triaangles), were measured for ROS activity ± 6 µM SHetA2. Vehicle is the SHetA2 solubilization buffer alone minus added drug or protein. Data shown as mean normalized fluorescence intensity at 597 nm across N = 7 measurements with the means ± SE denoted for each reaction condition, ns = not statistically significant, and **** = p < 0.0001.

The existence of an imbalance between prooxidant and antioxidant processes results in an increase in reaction oxygen species (ROS), which have been shown to act as a trigger and modulator of pathological events, that occur in DN (Miranda-Diaz et al., 2016). Generation of ROS by the mitochondrial electron transport system is partially responsible for kidney damage associated with DN (Miranda-Diaz et al., 2016; Brownlee, 2001). It is generally accepted that p66Shc shuttles to mitochondria to contribute to ROS production (Natalicchio et al., 2011; Pellegrini and Baldari, 2009). We tested whether SHetA2 could inhibit p66Shc-mediated ROS production. Treatment with SHetA2 resulted in statistically significant decrease of ROS production induced by both recombinant human p66Shc and CH2CB domain of human p66Shc (Figure 7). CH2CB is the N-terminal CH2 domain plus the small extension connecting into the phosphotyrosine binding domain (PTB). The current literature supports an argument that ROS produced by p66Shc is derived from the CH2CB domain and this domain is ROS active as a distinct protein from the full-length p66Shc. Our data suggest that there is an interaction between SHetA2 and the N-terminal domain of p66Shc.

## 3 Discussion

The kidney is the most important target of microvascular dysfunction in diabetes (Thomas et al., 2015). Currently, there is no specific cure or targeted therapy for DN. Angiotensin converting enzyme inhibitors and angiotensin receptor blockers have been the main stay of renal protection in diabetes for many years. Recently more drugs have added to the armamentarium of fighting chronic kidney disease, but none of the current drugs has been tested long enough to prove lasting protection for kidney tissue. Accordingly, there is still a strong need for novel therapeutics which will target mechanisms other than limiting proximal tubular exposure to filtered proteins. We conducted testing of small molecule SHetA2 in relevant animal model of type 1 diabetes. SHetA2 has potential for use as a prevention agent because it is orally-bioavailable and has minimal-to-no toxicity in preclinical studies (Benbrook, 2022) and an ongoing Phase 1 clinical trial (clinicaltrials.gov/NCT04928508). Our published data indicate that impairment of myogenic response to increased perfusion pressure in rats with hypertension-induced nephropathy is the result of increased p66Shc expression in renal microvessels (Miller et al., 2016) and that acute and chronic treatment with SHetA2 restores autoregulation of renal vessels (Miller et al., 2025). We have also previously shown that DN in STZ-induced, hyperglycemic SS rats is also mitigated by p66Shc as a result of increased K-_ATP_ channel activity and early onset hyperfiltration of glomerular afferent arterioles (Miller et al., 2018). In this study, we sought to explore whether SHetA2 also has the potential to mitigate DN injury through restoration of myogenic response and inhibition of K-_ATP_ channels via a p66Shc-specific mechanism.

It must be taken into consideration that even though SHetA2 modulates p66Shc activity, it certainly also has some p66Shc-independent effects. Targets of SHetA2 include mortalin, hsc70 and Grp78 (Benbrook, 2022). Our data using p66Shc knockout animals emphasize that seen effects upon renal vascular reactivity are mostly p66Shc-dependent. However, it may explain why some changes in calcium mobilization could be detected also in cells derived from p66Shc knockout rats.

As seen in Figure 1, SHetA2 appears to restore myogenic response not only in diabetic SS rats but also to some extent in control rats. The reason for this is that Dahl SS rats have vascular dysfunction at baseline. It was hypothesized that Dahl SS have an impaired myogenic response and constrictor response to ATP, due to decreased 20-HETE (Ren et al., 2014).

Analysis of SHetA2 effect upon p66Shc-mediated ROS production (Figure 7) indicated that it might act via preventing ROS production caused by CH2CB domain of p66Shc. The CH2CB domain of p66Shc interacts with cytochrome C in the mitochondria. The p66Shc-mediated response to oxidative stress is highly dependent on phosphorylation of serine 36 residue (Ser36), which is required for its transfer to mitochondria where it interacts with cytochrome C (Migliaccio et al., 1999). We have previously reported that p66Shc-S36A mutant, which is retained in cytoplasm, prevents p66Shc-mediated renal vascular dysfunction in rat model of DN (Miller et al., 2018).

**FIGURE 8:**
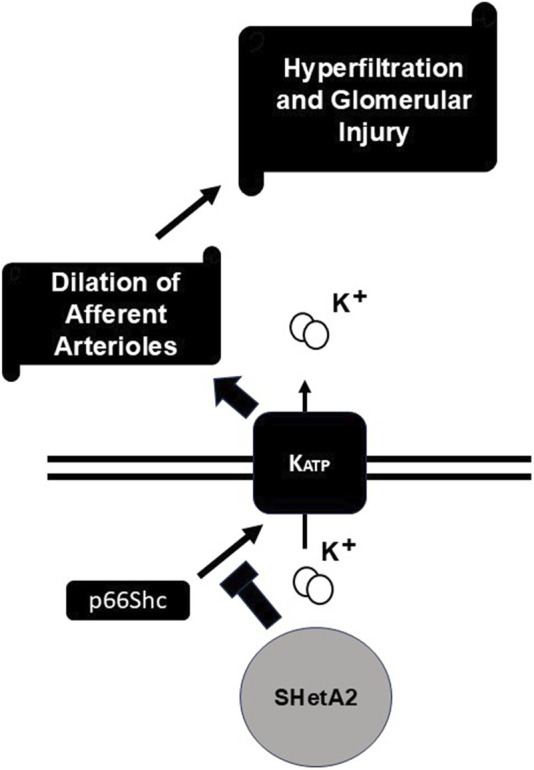
Proposed mechanism of SHetA2 action. P66Shc signaling promotes activation of K_ATP_ channels which result in undesirable dilation of afferent arterioles followed by hyperfiltration and glomerular injury. SHetA2 modulates p66Shc signaling and prevents K_ATP_ channels activation restoring microvascular reactivity of renal vessels and mitigating glomerular injury.

We tested whether targeting p66Shc could interfere with afferent arteriole dilation and glomerular hyperfiltration in rats with induced type 1 diabetes. Our data indicate that targeting p66Shc by either acute or chronic treatment with SHetA2 restored to some extent the renal afferent arteriolar reactivity blunted by DN. Moreover, our data suggest that SHetA2 interferes with p66Shc-mediated activation of K_ATP_ channels (Figure 8), since it did not increase calcium influx into SMC, but prevented glibenclamide-mediated effects. Despite SHetA2 ability to restore vascular reactivity in STZ-treated rats it had limited effect upon renal function, as measured by proteinuria, or on urinary NGAL, a marker of tubular injury. The reason for insufficient restoration of renal function could be based on the fact that prevention of hyperfiltration observed on the early onset of diabetes in insufficient to mitigate renal pathologies associated with diabetes. Disruption of podocyte function (Li et al., 2023), metabolic irregularities induced by hyperglycemia, mesangial proliferation could be principal factors causing renal damage (Dwivedi and Sikarwar, 2025). The restoration of renal vascular reactivity is an important factor in mitigation of renal damage but seems to be insufficient by itself to prevent pathological changes associated with diabetes.

## 4 Methods

### 4.1 Animals

Animal use and welfare procedures were followed according to the NIH Guide for the Care and Use of Laboratory Animals, and study protocols were reviewed and approved by the Medical College of Wisconsin Institutional Animal Care and Use Committee. The generation of genetically modified p66Shc-KO on the genetic background of Dahl SS was described previously (Slaughter et al., 2013). Male rats were weaned at 3 weeks of age and provided diet (Teklad 7034) and water *ad libitum*. At 9–10 weeks of age, rats were induced for hyperglycemia by single intraperitoneal injection of STZ (55 mg/kg). After 1 week, hyperglycemia was confirmed using nonfasting blood glucose measured using a meter and test strips (Contour; Bayer Healthcare). Only animals in which glucose levels exceeded 250 mg/dL throughout the 12-week induction period were used for analysis. To assess proteinuria, spot urine was collected from rats housed in metabolic cages for 1–2 h, 24 h after previous treatment. Urine was frozen at −80°C until analysis of total protein (Bradford assay, Bio-Rad, United States) and creatinine (BioAssay Systems, United States).

### 4.2 Administration of SHetA2

SHetA2 (NSC 726189) was supplied to Dr. Doris Benbrook by the US National Cancer Institute RAPID Program and processed for use in animal studies as described (Brosius et al., 2009). Once hyperglycemia was established, rats were given either vehicle control (30% Kolliphor HS15 w/v in water, 0.2% volume of body weight) or SHetA2/30% Kolliphor at 60 mg/kg by oral gavage with equivalent volumes. Oral gavage was performed 3 times weekly every other day except on weekends for a period of 12 weeks. Each week, fresh drug was bulk homogenized, aliquoted and sonicated on the day of treatment, and kept in the dark at 4°C between uses.

### 4.3 Afferent arteriolar responses

The perfused juxtamedullary nephron preparation was used to assess renal microvascular reactivity in rats, as described (Imig et al., 2008). In experiments involving acute treatment with SHetA2, the drug was applied 30 min prior to diameter recordings at 60 and 120 mmHg perfusion pressure.

### 4.4 Calcium measurements

Isolated renal vascular SMCs (Miller et al., 2016) (passage 6, 20,000 cells/dish) were seeded on 35 mm glass microwell dishes and serum-starved (0.5% fetal bovine serum in DMEM) for 24 h prior to experiments. Cells were loaded with Fluo4-AM (2 µM) at room temperature for approx. 1 h in the dark. HEPES (10 mM) was added to medium for dye loading. After dye loading, cells were washed twice with PSS (140 mM NaCl, 1.2 mM MgCl_2_, 4.5 mM KCl, 18 mM glucose, 10 mM HEPES, 2 mM CaCl_2_) then incubated in PSS containing DMSO (0.3%) or SHetA2 (0.25 µM, 1 μM, or 2.5 µM) for 35 ± 5 min. Cells were imaged on a Nikon A1r confocal microscope using a ×20 objective lens (NA = 0.75) and excitation/emission settings of 488/500–550 nm. Time lapse experiments were performed at room temperature. Baseline fluorescence was recorded for 1 min prior to stimulation with ET-1 (100 nM). Changes in FL were recorded for approx. 7 min after stimulation. Images were analyzed using ImageJ FIJI software and calculations were performed in MS Excel. Changes in Ca^+2^ are reported as changes in FL compared to baseline FL levels (ΔF/F_0_). Total cytosolic Ca^+2^ was calculated as the integral under each curve and is reported in arbitrary units (AU).

### 4.5 Western blotting

Urine collected from non-diabetic, diabetic and SHetA2-treated diabetic rats at 12-0weeks treatment was diluted in water and boiled in 1X sample buffer for 5 min. Sample volumes (up to 10 μL per lane) were normalized to urine creatinine levels and separated on 4%–15% SDS-Page gels and treansferred onto PVDF membranes (Bio-Rad). Membranes were blocked with Intercept TBS buffer (LICOR), probed using antibodies against rLipocain-2 (NGAL) (1:2000; R&D Systems, Minneapolis, MN). Blots were subsequently probed with donkey anti-goat horseradish peroxidase (HRP), secondary antibody (1:10,000, Santa Cruz). Chemiluminescence was measured using the Bio-Rad ChemiDoc MP system after exposure of the blots to SuperSignal West Femto ECL detection reagents (Thermo Scientific) for 5 min.

### 4.6 ROS measurement

Protein expression (human p66Shc and CH2CB) and hydrdoethidine fluorescent assays using an ISS photon counting spectrofluorometer were performed as previously described (Haslem et al., 2022). SHetA2 was solubilized in 20 mM MES pH 5.5, 100 mM NaCl, and 50% w/v PEG 400 at a final concentration of 30 μM and added to reaction mixtures at a final concentration of 6 μM. When present, final protein concentrations were 10 uM. SHetA2 stocks were prepared fresh, and reaction mixtures protected from ambient light. Each assay condition was repeated 7 times using 3 different protein preparations for each target protein (first preparation for each protein has 3 technical replicates). Average values were used for statistical analysis.

### 4.7 Statistical methods

For vessel reactivity to ATP a two-way repeated measures ANOVA was used. For vessel reactivity to pressure, a Mann-Whitney two-tailed t-test was performed. All tests were performed using GraphPad Prism 10.2.3. For total calcium measurements, Kruskal-Wallis tests and post-hoc Dunn’s Test were performed using R.

## Data Availability

The raw data supporting the conclusions of this article will be made available by the authors, without undue reservation.
